# Retraction Note: Tamoxifen suppresses brain metastasis of estrogen receptor-deficient breast cancer by skewing microglia polarization and enhancing their immune functions

**DOI:** 10.1186/s13058-025-02042-5

**Published:** 2025-05-12

**Authors:** Shih-Ying Wu, Sambad Sharma, Kerui Wu, Abhishek Tyagi, Dan Zhao, Ravindra Pramod Deshpande, Kounosuke Watabe

**Affiliations:** Department of Cancer Biology, Wake Forest Baptist Medical Center, Wake Forest University School of Medicine, Winston-Salem, NC 27157 USA

**Breast Cancer Research (2021) 23:35**

10.1186/s13058-021-01412-z

The Editor-in-Chief has retracted this article. After publication, concerns were raised regarding highly similar images in Fig. 1a (OVX + E2 mouse 3) of this article and Fig. 6l (Veh mouse 1) of [1].

The authors have stated that the OVX + E2 mouse 3 images was incorrect, but have been unable to provide the full raw data from this experiment upon request. The Editor-in-Chief therefore no longer has confidence in the presented data.

Shih-Ying Wu, Abhishek Tyagi, Dan Zhao, Ravindra Pramod Deshpande and Kounosuke Watabe disagree with this retraction. Sambad Sharma and Kerui Wu did not respond to any correspondence from the Publisher about this retraction.
